# Das Anti-Synthetase-Syndrom: Eine Sonderform der Myositis mit vielen Gesichtern

**DOI:** 10.1007/s00739-022-00835-3

**Published:** 2022-08-17

**Authors:** Kastriot Kastrati, Helga Lechner-Radner, Ellen Gelpi

**Affiliations:** 1 Universitätsklinik für Innere Medizin III, Klinische Abteilung für Rheumatologie, Medizinische Universität Wien, Währinger Gürtel 18–20, 1090 Wien, Österreich; 2 Abteilung für Neuropathologie und Neurochemie, Universitätsklinik für Neurologie, Medizinische Universität Wien, Wien, Österreich

**Keywords:** Aminoacyl-tRNA Synthetase, Interstitielle Lungenerkrankung, Arthritis, Myositis-Autoantikörper, Muskelbiopsie, Aminoacyl-tRNA synthetase, Interstitial lung disease, Arthritis, Myositis autoantibodies, Muscle biopsy

## Abstract

Das Anti-Synthetase-Syndrom (ASyS) ist eine klinisch heterogene und seltene Autoimmunerkrankung, in der es zur Bildung von Autoantikörpern gegen Aminoacyl-tRNA-Synthetasen kommt. Klinisch findet man klassischerweise die Trias aus Myositis, Arthritis und prognosebestimmender interstitieller Lungenerkrankung. Wir berichten über einen 30-jährigen Patienten mit rezidivierenden Fieberschüben, symmetrischen Gelenkschwellungen, Muskelbeschwerden und progredienter Belastungsdyspnoe, bei dem in Zusammenschau des Nachweises von Anti-Jo-1-Antikörpern die Diagnose eines ASyS gestellt wurde. Unter einer Kombinationstherapie aus Glukokortikoiden und Azathioprin konnte eine Remission der Myositis und Arthritis sowie eine Regredienz der ILD-assoziierten Veränderungen im Thorax-CT erzielt werden. Die frühzeitige Erkennung der Erkrankung und die Bestimmung myositisspezifischer Antikörper sind für die Diagnostik und Prognostik der Erkrankung von zentraler Bedeutung. Betroffene Patient:innen können dadurch rasch einer adäquaten, auf ihre Organmanifestation abgestimmten Therapie zugeführt werden.

## Abstract (en)

Antisynthetase syndrome (ASyS) encompasses a clinically heterogeneous and rare autoimmune disease characterized by the production of autoantibodies against aminoacyl-tRNA synthetases. Clinically, the disease presents with the triad of myositis, arthritis, and interstitial lung disease. We report the case of a 30-year-old man with recurrent episodes of fever, symmetric joint swelling, muscle symptoms, and progressive exertional dyspnea, in whom the diagnosis of ASyS was made based on the detection of anti-Jo‑1 antibodies. The combination therapy of glucocorticoids and azathioprine resulted in remission of the myositis and arthritis as well as regression of the ILD-associated changes in the chest CT. Early recognition of the syndrome and the assessment of myositis-specific antibodies are nowadays essential for an accurate nosological classification and definition of therapy strategies tailored to the severity of organ involvement.

## Einleitung

Das Anti-Synthetase-Syndrom (ASyS), als Teil der idiopathischen inflammatorischen Myopathien bzw. Myositiden, ist eine seltene und heterogene Systemerkrankung. Die Erkrankung ist mit einer hohen Morbidität behaftet und zeichnet sich durch den serologischen Nachweis von myositisspezifischen Autoantikörpern gegen Aminoacyl-Transfer-RNA-Synthetasen (Aminoacyl-tRNA-Synthetasen) aus [1]. Die Familie der tRNA-Synthetasen besteht aus konservativen zytoplasmatischen und mitochondrialen Enzymen, welche die Translationsprozesse und Proteinbiosynthese steuern. Neben dieser Hauptfunktion sind die Aminoacyl-tRNA-Synthetasen an der Entwicklung von Immunantworten, der Regulation der Transkription und der genspezifischen Stummschaltung der Translation beteiligt.

Während des letzten Jahrzehnts wurden diese Proteine mit neurologischen Störungen, Infektionsreaktionen, Malignität und Autoimmunerkrankungen, einschließlich dem ASyS, in Verbindung gebracht. 1980 wurde erstmals Anti-Jo‑1 aus der Familie der Anti-Synthetase-Antikörper identifiziert. Mittlerweile erfolgte die Entdeckung weiterer sieben Antikörper (gegen PL7, PL12, EJ, OJ, KS, Ha und Zo). Das klinische Spektrum des ASyS ist äußerst versatil und umfasst neben der interstitiellen Lungenerkrankung (ILD) eine entzündliche Affektion der Skelettmuskulatur (Myositis) bzw. selten auch des Myokards (Myokarditis), eine meist nicht erosive Arthritis, ein Raynaud-Phänomen, Fieber und hyperkeratotische Hautveränderungen an den Fingern („Mechanikerhände“) [2, 8]. Die mit dem Krankheitsbild assoziierte interstitielle Lungenerkrankung (ILD) ist mit 69–100 % eine hochprävalente klinische Manifestation und wichtiger Faktor für Morbidität sowie Mortalität, weswegen sich die Therapie hauptsächlich nach dem Vorhandensein und Schweregrad der ILD richtet [3].

Interstitielle Lungenerkrankung, Myositis und Arthritis sind die klassische Trias

Histologische Merkmale der Myositis im Rahmen des ASyS sind perifaszikuläre Muskelfaserveränderungen mit perifaszikulären Nekrosen und Atrophien sowie eine perifaszikuläre HLA-Klasse-I-Expression. Begleiterscheinend sind zumeist perimysiale Zellinfiltrate ersichtlich. Im Vergleich zur Polymyositis sind beim ASyS endomysiale Zellinfiltrate untypisch [4, 5]. Für das Anti-Synthetase-Syndrom sind gegenwärtig Diagnosekriterien nicht existent und mangels randomisierter bzw. kontrollierter Studien fundiert das Management auf Fallserien und Expertenmeinungen. Neben Glukokortikoiden kommen unter anderem Mycophenolat mofetil, Azathioprin, Calcineurin-Inhibitoren und Rituximab zum Einsatz [6].

Zum Screening auf Organbeteiligung und Definition des individuellen Phänotyps werden beim ASyS ein Assessment aus hochauflösender Computertomographie (hr-CT) des Thorax, Lungenfunktionsuntersuchung, Erhebung der Kreatinkinase (CK) im Labor und eine sorgfältige Muskelkraftuntersuchung empfohlen [3]. Anhand dieses exemplarischen Fallberichts sollen Organscreening und therapeutisches Management für die klinische Praxis nähergebracht werden.

## Fallbericht

Zur stationären Aufnahme gelangte ein 30-jähriger Patient, der von einem Peripheriespital aufgrund seit etwa vier Wochen bestehender rezidivierender Fieberschübe, trockenem Reizhusten, progredienter Belastungsdyspnoe, geschwollenen Fingergelenken, Muskelschmerzen und -schwäche der proximalen Oberarme und -schenkeln an die Universitätsklinik für Rheumatologie transferiert wurde. Bei leicht reduziertem Allgemeinzustand, mit 37,5 °C subfebril und klarem Bewusstseinszustand imponierte der Patient im Status muskelgeschwächt in den proximalen Aspekten der oberen und unteren Extremitäten. Des Weiteren zeigte sich eine symmetrische Arthritis mit schmerzhafter, synovitischer Schwellung der Handgelenke, Metakarpophalangealgelenke und proximalen Interphalangealgelenke. In der Auskultation der Lunge konnte beidseitig ein basales inspiratorisches Knisterrasseln erhoben werden.

Mit den muskuloskelettalen Beschwerden des Patienten kompatibel fanden sich laborchemisch, im Sinne einer Muskelentzündung, erhöhte Muskelenzyme mit einer Kreatinkinase (CK) von 4816 U/l (Normalwert: < 190 U/l) und ein Transaminsenanstieg mit einer Glutamat-Oxalacetat-Transaminase (GOT) von 230 U/l (Normalwert: < 50 U/l) sowie Glutamat-Pyruvat-Transaminase (GPT) von 260 U/l (Normalwert: < 50 U/l), bei normwertiger Gamma-Glutamyl-Transferase (GGT). Die erhöhte Blutsenkungsgeschwindigkeit (BSG) von 30 mm in der ersten Stunde und ein moderat erhöhtes C‑reaktives Protein (CRP) von 1,8 mg/dl waren kompatibel mit dem systemisch-inflammatorischen Charakter des vorliegenden Krankheitsbildes. Die ausgeprägte Muskelschwäche mit besonderer Betonung der Oberschenkel konnte mit dem für inflammatorische Myopathien validierten Manual-Muscle-Test (MMT) objektiviert werden, wo der Patient 46 von 80 erzielbaren Punkten erreichte.

**Figure 1:**
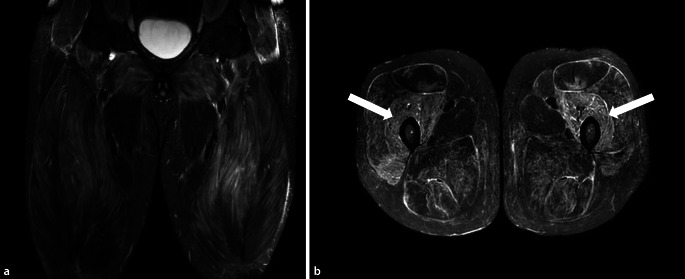

**Figure 2:**
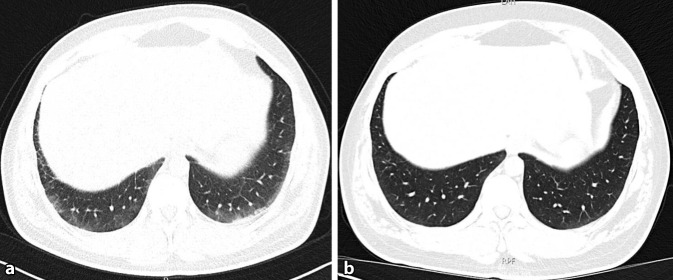

Als morphologisches Korrelat fand sich in der Magnetresonanztomographie (MRT) der unteren Extremitäten ein ausgeprägtes Muskelödem in sämtlichen abgebildeten Muskelgruppen beider Oberschenkel sowie auch der Glutealmuskulatur (Abb. 1). In Anbetracht des pathologischen Auskultationsbefundes wurde eine hochauflösende Computertomographie (hr-CT) des Thorax veranlasst, in der ein unterlappenbetontes symmetrisches dorsales Milchglas, in erster Linie einer nichtspezifischen interstitiellen Pneumonie (NSIP) entsprechend, detektiert wurde (Abb. 2a). Lungenfunktionell fand sich die Diffusionskapazität mittelgradig eingeschränkt.

In 15–30 % der Fälle sind Husten und Dyspnoe die initialen Beschwerden

Zur weiteren differenzialdiagnostischen Abklärung der Belastungsdyspnoe erfolgte eine Troponin-T-hochsensitiv-Bestimmung, die mit 380 ng/l zwar eine Auslenkung ergab, aber in den seriellen Kontrollen ohne relevante Dynamik blieb. Mit der Fragestellung einer myokardialen Beteilung wurde ein 1,5-T-MRT des Herzens durchgeführt. Diese ergab zwar keinen Nachweis einer Myokarditis bzw. eines Myokardödems oder Late-Enhancements, allerdings einen diskreten Perikarderguss ohne assoziierte Perikarditis.

Im Hinblick auf die Arthritis mit Charakter einer rheumatoiden Arthritis konnten durch konventionelles Röntgen destruierende bzw. erosive Gelenkschäden ausgeschlossen werden. Der Patient negierte raynaudtypische Hautveränderungen. Ebenso konnte durch eine unauffällige Kapillarmikroskopie eine Mikroangiopathie ausgeschlossen werden. In Zusammenschau der Befunde (interstitielle Lungenerkrankung im Sinne eines NSIP-Musters, symmetrische Polyarthritis, Fieber und Myositis) wurde die Verdachtsdiagnose eines Anti-Synthetase-Syndroms gestellt, welche sich durch den hochtitrigen Nachweis von Antikörpern gegen Jo‑1 von 166 U/ml (negativ: < 7 U/ml) im Serum bestätigen ließ.

Eine myokardiale Beteiligung beim ASyS ist selten

Zur histologischen Diagnosesicherung wurde ferner auf Basis des in der MRT entzündlich affektierten M. vastus lateralis links komplikationslos eine Biopsie entnommen. Nach der klinischen Diagnosestellung und histologischen Probenentnahme wurde eine immunsuppressive Therapie mit 75 mg Prednisolon per os (1 mg/kgKG) initiiert. Unter der Steroidmedikation trat ein rascher und signifikanter Rückgang der Muskelenzyme (nach 3 Tagen betrug die CK 1980 U/l) sowie Normalisierung der Transaminasen ein. Auch präsentierte sich der Patient klinisch wenige Tage nach Therapiebeginn beschwerdefrei und afebril, sodass das weitere Management im ambulanten Setting erfolgte. Bei langsamer Steroidreduktion (10 mg pro Woche) fand sich der Patient 6 Wochen nach dem stationären Aufenthalt in der ambulanten Kontrolle weiterhin kardiorespiratorisch sowie muskulär beschwerdefrei.

Das bei Entlassung empfohlene Muskeltraining unter Einhaltung der individuellen Belastungsgrenzen über ein niedergelassenes physiotherapeutisches Zentrum sowie sportliche Aktivität mit Schwerpunkt auf Schwimmen und Radfahren verbesserten nach Angaben des Patienten die muskuläre Kondition zusehends. Rekurrente Fieberschübe wurden negiert. Im Status fanden sich keine synovitisch geschwollenen Gelenke oder kutane Veränderungen. Laborchemisch hatten sich die Muskelenzyme und Akut-Phase-Parameter (BSG und CRP) normalisiert.

**Figure 3:**
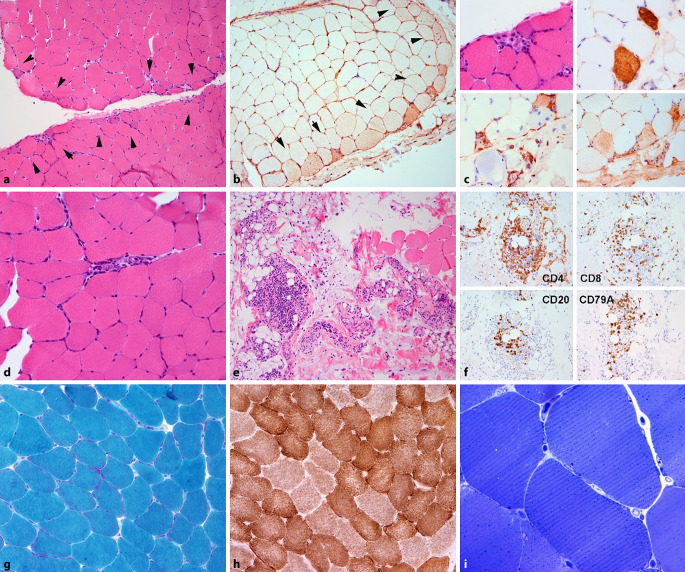

In der Zwischenzeit lag das histologische Ergebnis der Muskelbiopsie vor, die das Bild einer floriden Myositis mit charakteristischen histopathologischen Merkmalen einer ASyS assoziierten Myositis ergab: Bei Schwerpunkt der Entzündung des Perimysiums bzw. perifaszikulär war die inflammatorische Pathologie durch CD3-, CD4 sowie CD8-positive T‑lymphozytäre und geringe CD20- und CD79A-positive B‑Zellinfiltrate im subperimysialen Endomysium, im Perimysium und im angrenzenden Bindegewebe gekennzeichnet. Begleitend imponierten auch perifaszikulär akzentuierte Einzelfasernekrosen und Faserregenerate sowie eine HLA-Klasse-I-Antigen-Expression. Zudem fanden sich Ablagerungen des terminalen Komplementkomplexes an den Membranen einzelner Muskelfasern, vor allem aber in den Muskelfasernekrosen, nicht jedoch in den Kapillaren. Diese zeigten eine reguläre Dichte. Es zeigten sich keine mitochondrialen Alterationen. Abb. 3 liefert eine Darstellung der histopathologischen Merkmale des affektierten Muskels.

Als cortisoneinsparende Therapie standen, insbesondere unter Berücksichtigung der Lungenbeteiligung, unter anderem Mycophenolat mofetil, Calcineurininhibitoren, Azathioprin und Rituximab als therapeutische Optionen zur Diskussion. In Anbetracht der COVID-19-Pandemie und der erhöhten Hospitalisations- und Mortalitätsrate bei SARS-CoV-2-infizierten Patienten mit rheumatischen Erkrankungen einerseits und der Eignung von Rituximab eher als 2. Linienoption im Falle einer Progredienz der ILD andererseits wurde von Rituximab abgesehen. Aufgrund des aktiven Kinderwunsches und einer bevorstehenden Familienplanung wurde Azathioprin gegenüber Mycophenolat mofetil und Calcineurininhibitoren vorgezogen. Unter dem Therapieregime aus Azathioprin (2 mg/kgKG) und graduellem Cortison-Reduktionsschema wurde eine Verlaufsuntersuchung der ILD mittels hr-CT veranlasst. Im Vergleich zur Voruntersuchung bei Erstmanifestation fand sich nun eine deutliche Regredienz der vorbeschriebenen subpleural betonten Milchglasveränderungen.

## Schlussfolgerung

Das Anti-Synthetase-Syndrom (ASyS) ist eine erworbene und heterogene Erkrankung, die durch den Nachweis von Autoantikörpern gegen tRNA-Synthetasen und Trias aus ILD, Myositis und Arthritis charakterisiert ist. Die Lungenbeteiligung ist mit erhöhter Morbidität und Mortalität vergesellschaftet, weswegen eine möglichst frühe Diagnosestellung und Therapieeinleitung essenziell sind. Immunsuppressive Therapiestrategien basieren aufgrund mangelnder Studien auf niedrigem Level der Evidenz, sind jedoch unabdingbar zur erfolgreichen Kontrolle der Krankheitsaktivität.

## Fazit für die Praxis

- Als Myositis-Subtyp mit dominierender Lungenbeteiligung liegt die ILD-Prävalenz bei 69–100 %. Die Abklärung einer pulmonalen Beteiligung ist daher bei jedem Patienten indiziert.
- Die Bestimmung der 8 verfügbaren Anti-Aminoacyl-tRNA-Synthetase-Autoantikörper ist für die Diagnosestellung, die Einschätzung der Prognose und des Therapieansprechens essenziell.
- Neben Cortison gehören Mycophenolat mofetil, Azathioprin, Calcineurininhibitoren und Rituximab zum therapeutischen Armamentarium.
- Kreatinkinase (CK) dient als Indikator der Muskelbeteiligung und als Parameter zur Beurteilung der Krankheitsaktivität. Mittels MRT der Muskulatur kann sowohl die Myositis dargestellt als auch ein Zielmuskel für eine Biopsie identifiziert werden. Diese sollte auch umgebendes Bindegewebe miterfassen.
